# SALDI-MS and SERS Multimodal Imaging: One Nanostructured Substrate to Rule Them Both

**DOI:** 10.1021/acs.analchem.1c04118

**Published:** 2022-02-01

**Authors:** Stefania-Alexandra Iakab, Gerard Baquer, Marta Lafuente, Maria Pilar Pina, José Luis Ramírez, Pere Ràfols, Xavier Correig-Blanchar, María García-Altares

**Affiliations:** † Department of Electronic Engineering, Rovira i Virgili University, Tarragona 43007, Spain; ‡ Spanish Biomedical Research Centre in Diabetes and Associated Metabolic Disorders (CIBERDEM), Madrid 28029, Spain; § Instituto de Nanociencia y Materiales de Aragón (INMA), CSIC-Universidad de Zaragoza, Zaragoza 50009, Spain; ∥ Departamento de Ingeniería Química y Tecnologías del Medio Ambiente, Universidad de Zaragoza, Campus Río Ebro-Edificio I+D+i, C/Mariano Esquillor s/n, Zaragoza 50018, Spain; ⊥ Networking Research Center on Bioengineering, Biomaterials and Nanomedicine, CIBER-BBN, Madrid 28029, Spain; # Institut d’Investigació Sanitària Pere Virgili (IISPV), Reus 43204, Spain

## Abstract

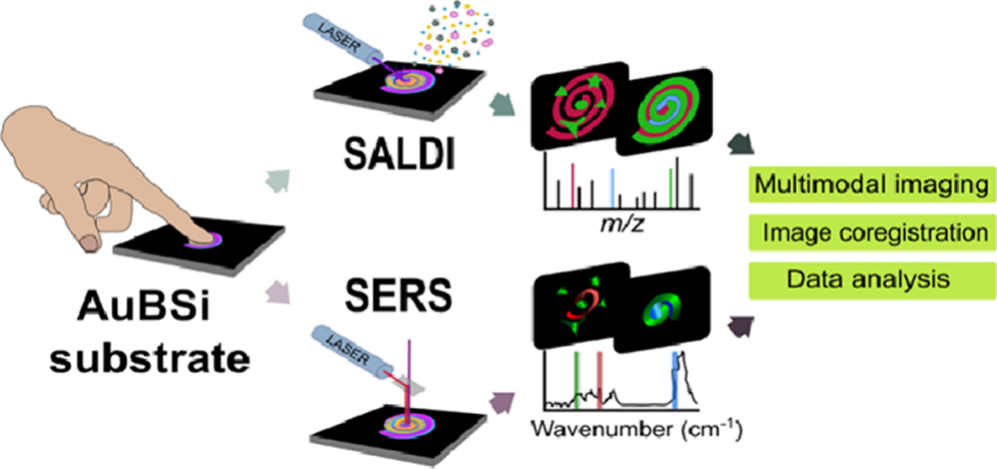

Imaging techniques based on mass spectrometry or spectroscopy methods inform *in situ* about the chemical composition of biological tissues or organisms, but they are sometimes limited by their specificity, sensitivity, or spatial resolution. Multimodal imaging addresses these limitations by combining several imaging modalities; however, measuring the same sample with the same preparation using multiple imaging techniques is still uncommon due to the incompatibility between substrates, sample preparation protocols, and data formats. We present a multimodal imaging approach that employs a gold-coated nanostructured silicon substrate to couple surface-assisted laser desorption/ionization mass spectrometry (SALDI-MS) and surface-enhanced Raman spectroscopy (SERS). Our approach integrates both imaging modalities by using the same substrate, sample preparation, and data analysis software on the same sample, allowing the coregistration of both images. We transferred molecules from clean fingertips and fingertips covered with plasticine modeling clay onto our nanostructure and analyzed their chemical composition and distribution by SALDI-MS and SERS. Multimodal analysis located the traces of plasticine on fingermarks and provided chemical information on the composition of the clay. Our multimodal approach effectively combines the advantages of mass spectrometry and vibrational spectroscopy with the signal enhancing abilities of our nanostructured substrate.

## Introduction

Label-free imaging techniques are crucial for understanding biological mechanisms at a molecular level. They are used for investigating a wide range of issues such as plant-based renewable energy, microbiological assays, diseases (in clinical medicine), and even forensic specimens.^1−5^ Vibrational spectroscopy and mass spectrometry imaging techniques are the most popular choices to specifically and simultaneously map a wide range of molecules present in living organisms or frozen tissues.^1^

Raman imaging is frequently used for exploring the chemical composition of biological samples.^6^ It offers high spatial resolution maps (down to ∼250 nm lateral resolution) with information about the molecular structure (secondary structure of proteins, saturation level of lipids, etc.)^7^ but with limited sensitivity and specificity. Surface-enhanced Raman spectroscopy (SERS) boosts Raman sensitivity and specificity through electromagnetic enhancement provided by plasmon resonances in the metal substrate, that is, the Raman signals of molecules in the close vicinity of metallic nanostructures are amplified by several orders of magnitude, and through chemical enhancement when a charge-transfer mechanism in the metal–adsorbate complex is established.^8^ Gold and silver nanoparticles are popular for SERS detection, as they are stable in air and can be used over a wide range of laser wavelengths (400–1000 nm for Ag and 600–1200 nm for Au).^9−12^ SERS is frequently used not only for body fluid analyses, such as pathogen detection (e.g., bacteria from urine and blood^11^) but also for imaging applications such as tumor margin determination,^10^ single-cell analysis,^12^ and even for revealing chemical information from latent fingermarks.^9^ However, sample preparation for SERS imaging using metallic nanoparticles is complicated (nanoparticles need to be functionalized with labels and binding molecules for a specific target),^13,14^ while label-free experiments often experience nanoparticle surface saturation.^15^

Matrix-assisted laser desorption/ionization mass spectrometry imaging (MALDI-MSI) is commonly used in proteomics and metabolomics studies^16^ as it offers rich, high-quality spectra, with specific chemical information (such as molecular weight and isotopic pattern), and even tandem mass spectrometry information (MS2) that makes the identification of molecules possible.^17^ However, MALDI has limitations: the ions from the organic matrices generate noise in the low mass range, the spatial resolution is lower compared to Raman imaging (often down to ∼20 μm, due to the heterogeneous co-crystallization of the matrix and analyte, and delocalization of analytes caused during sample preparation), and sometimes the mass analyzer is not sensitive enough. Surface-enhanced laser desorption/ionization mass spectrometry imaging (SALDI-MSI) uses nanostructured surfaces often made of gold, silver, and silicon^18−20^ to promote the desorption and ion formation of small molecules; this eliminates the limitations of the matrix and enables imaging at higher lateral resolutions with cleaner spectra.^19^ For instance, nanostructured silicon surfaces have various applications for SALDI imaging: when embedded with siloxane compounds, they can characterize peptide microarrays, single cells, tissues, blood, and urine,^21^ and when decorated with gold nanoparticles they can selectively detect metabolites from tissue surfaces.^22^ However, the most popular mass spectrometers used for imaging are still limited to spatial resolutions >10 μm. SALDI also presents some limitations: (1) SALDI sample preparation using nanoparticles is often nor reproducible due to their aggregation,^23^ (2) there are few commercial SALDI substrates on the market, and (3) SALDI substrate fabrication is a complex process that requires specific technology and knowledge, which is not available to all laboratories. Thus, MALDI-MSI is still the technique of choice for many laboratories, as the analytical workflows are validated and standardized, and there is a variety of instrumentation designed for MALDI, both from the point of view of sample preparation (e.g., matrix sprayers) and image acquisition (e.g., target holders designed to hold ITO slides).

Molecular imaging data are described by two keywords: spectral information and lateral resolution. As a result, MALDI and Raman have been combined as a multimodal imaging approach to encompass the best of both worlds.^24,25^ The main advantage of multimodal imaging is the complementarity of the two techniques: the high spatial resolution images from Raman and the rich spectral information from MALDI. For example, correlation features between MSI, Raman, and IR images allowed the characterization of lipids from hamster brains in terms of species identification (MSI) and located the areas of the brain that are protein-rich in α-helical and β-sheet secondary structures (Raman and IR).^26^ Unfortunately, sample preparation, image acquisition, and data processing workflows are challenging for multimodal imaging. The use of different substrates and sample preparation protocols as well as the challenges to measure the same sample are still issues to be addressed^24^ (Table S1). For example, groups that used the same substrate (ITO glass slide) to perform Raman imaging and MALDI-MSI had to add an additional step while preparing the sample for MSI analysis: organic matrix deposition for MALDI^27,28^ or an unconventional silicon-based nanoparticle system for SALDI.^29^ Multimodal data processing also struggles with different file formats and different data preprocessing (Table S1). For instance, MALDI spectra need to be aligned and binned while Raman spectra need to be cleaned from cosmic rays and have their baseline corrected.^30,31^ These steps are often performed with software provided by instrument manufacturers or developed in-house.^24^ Nevertheless, there is no standardized workflow for either sample preparation or data processing in multimodal imaging.

We created a multimodal imaging approach to acquire molecular images by surface-enhanced Raman and MSI, that is, SERS and SALDI-MSI, respectively, from the same sample using the same nanostructured silicon substrate with a one-step sample preparation method. To demonstrate our approach, we stamped a clean fingertip and a fingertip covered by residues of plasticine modeling clay onto our nanostructured substrate. SERS and SALDI imaging revealed *in situ* the complex molecular information of fingermarks described not only by endogenous compounds (secreted by the eccrine and sebaceous glands) but also by exogenous (plasticine) molecules at a micron-scale resolution.

## Materials and Methods

### Multimodal Imaging Substrate

Pristine n-type silicon wafers from MicroChemicals GmbH (Ulm, Germany) were used in a reactive ion etching chamber to obtain the black silicon. The high purity grade gold target was obtained from Kurt J. Lesker Company (Hastings, England) and used in a magnetron sputtering chamber to deposit gold on the black silicon and obtain the final substrate labeled AuBSi. A more detailed fabrication process is described in our previous work.^22^ Morphological characterization of the substrate was done with a field-emission scanning electron microscope equipped with a focused ion beam from Thermo Scientific, model Scios2; reflectance measurements were carried out with a Lambda-950 spectrophotometer, equipped with deuterium and tungsten lamps; the surface roughness was characterized by an atomic force microscope; and the hydrophobic behavior of the substrate was determined by measuring the contact angle of Milli-Q water droplets using a tensiometer together with Oneattension software (Biolin Scientific).

### Sample Preparation

The same sample preparation was used for both SALDI MSI and SERS imaging acquisitions. For drawing the pattern using inks, 2 mg of rhodamine 6G and malachite green (MG) (Sigma-Aldrich) were diluted in two separate 10 mL of butanol/terpineol (1:1) solutions. These solutions were sonicated for 15 min and were printed on the AuBSi substrate using a Dimatix DMP-2850 materials printer equipped with a DMC-11610 cartridge (Fujifilm Dimatix Inc., Santa Clara, CA, USA). 13 layers of two partially overlapping squares (one square printed with a R6G solution and the other with MG solution) were drawn to assess the specificity and lateral resolution capabilities of each imaging technique. For substrate evaluation, the AuBSi substrate was immersed in different concentrations of R6G and MG in ethanol (from 1 μM to 1 mM). In the case of fingermark imaging, the clean and stained fingermarks were obtained from a volunteer working in the laboratory. The clean fingermark was obtained after rubbing the finger with a disinfectant alcoholic hydrogel and the stained fingermark (from the same finger) was obtained after playing with modeling clay (or plasticine) model Art. 70 JOVI in green color. Molecules from the same finger (first clean then stained) were transferred onto the nanostructured substrate by lightly pressing the finger on the surface of the AuBSi substrate for 10 s. The stained finger’s surface was inspected for cleanliness prior to imprinting to avoid transferring the plasticine residue.

### Image Acquisitions

MSI data were acquired using a MALDI TOF/TOF UltrafleXtreme instrument with SmartBeam II Nd:YAG/355 nm laser from Bruker Daltonics. Acquisitions were carried out in the 50–1200 Da range in the reflectron mode, positive ionization mode using the large laser spot size settings, operated at 2 kHz, and collecting a total of 1000 shots per pixel. The negative ionization mode was used only for the measurement represented in Figure S4A. Specific acquisition parameters:

- Rhodamine immersion: 1 × 0.8 mm^2^ (before SERS measurement) and 0.8 × 0.8 mm^2^ (after SERS measurement) at 20 μm lateral resolution
- Inkjet-printed Raman reporters:
  Positive mode: 3.1 × 1 mm^2^ at 30 μm lateral resolution
  Negative mode: 4 × 2.5 mm^2^ at 250 μm lateral resolution
- Clean and stained fingermarks: 3.1 × 3 mm^2^ (clean) and 4.4 × 4 mm^2^ (stained) at 50 μm lateral resolution

SERS/Raman measurements were performed on a Renishaw inVia confocal Raman microscope. All maps were recorded at room temperature with a 633 nm laser, 1200 l/mm grating optical lens 50× with NA 0.75, and 1 s of integration time, unless otherwise indicated. For measurements illustrated in Figure S5, we used 514, 633, and 785 nm excitation wavelengths using the 1200 l/mm grating for the 633 and 785 nm lasers and the 2400 l/mm grating for the 514 nm laser and the same parameters as before. Specific acquisition parameters.

- R6G immersion:
  Figure 2: 2.2 × 2.1 mm^2^ area at 20 μm lateral resolution
  Figure S7: (D—before SALDI) 3 × 3 mm^2^ area and (E—after SALDI) 1 × 2 mm^2^ area at 20 μm lateral resolution
- Inkjet-printed Raman reporters: overlapping squares areas from
  Figure 3D: 0.7 × 0.6 mm^2^ area at 5 μm lateral resolution; 2 s integration time.
  Figure 3E: 0.35 × 0.23 mm^2^ area at 1 μm lateral resolution; 2 s integration time
  Figure S5: 0.32 × 0.24 mm^2^ area at 20 μm lateral resolution
- Clean fingermark: 0.8 × 0.7 mm^2^ area at the 5 μm lateral resolution
- Stained fingermark: 0.75 × 1 mm^2^ area at the 7 μm lateral resolution

### Data Preprocessing and Visualization

The raw SERS images were preprocessed using WiRE 5 software (from Renishaw) following basic chemometric algorithms: baseline correction (polynomial fitting), cosmic ray removal (software built-in algorithms Nearest Neighbor and Width of Features), and smoothing (Savitzky–Golay). Saturated spectra were zapped during preprocessing. Spectral axis alignment was done before each map acquisition by adjusting the silicon peak to a 520.5 cm^–1^ Raman shift. The preprocessed data sets were exported into *.txt files and converted into the imzML format using the open-source Raman2imzML R package.^32^ The converter package uses the spectral and spatial information to create an imzML file, the standard imaging file format used in the mass spectrometry community. This step is necessary to visualize both Raman and MS images with the same software. All SERS images were visualized without normalization.

The raw SALDI MSI data were exported into imzML files and processed using rMSIproc,^33^ an open-source software specifically designed to efficiently handle large MSI data sets. Spectral smoothing was done using Savitzky–Golay, mass calibration was based on the linear or loess mass error fitting model using the Au cluster peaks as internal references, and the peak matrix was created after peak picking and binning. All processing parameters were the default rMSIproc settings, unless otherwise mentioned. All ion images were normalized by TIC.

### Image Coregistration Protocol

Image coregistration was performed using a set of R and MATLAB in-house scripts available under the General Public Licence v3.0 at https://github.com/gbaquer/rMSIcoregistration. A single image (corresponding to a band in SERS or an ion in SALDI) was manually selected for each data set to represent a distinct morphology. The R package RNiftyReg available at CRAN^34^ was used to automatically register these representative images in the ink data sets used during optimization. The highly complex morphologies presented by the fingermark data sets required manual registration using “teaching points”. MATLAB functions “cpselect” (selection of point pairs) and “fitgeotrans” (generation of the transformation matrix) were used for this purpose as, unlike any available R counterpart, they provide a user-friendly GUI. The “affine” transformation type was used in both cases. Regardless of the registration type (automatic or manual) RNiftyReg was used to transform all data sets (SERS into SALDI and vice versa). The result is an R vector linking each pixel in the SERS data set to its closest counterpart in the SALDI data set and vice versa. This result is fully compatible with the rMSI data set class (rMSIprocPeakMatrix^33^) and it enables downstream multimodal analysis (such as transforming *k*-means clustering from one technique to the other). A diagram of the complete coregistration protocol is shown in Figure S10.

The putative identification of *m*/*z* values was done using the human metabolome database HMDB,^35^ Metlin,^36^ with 0.1 Da error permitted due to the poor spectral resolution and mass calibration in the low mass range.

## Results and Discussion

### Optimization Using Inks

#### AuBSi Substrate

**Figure 1:**
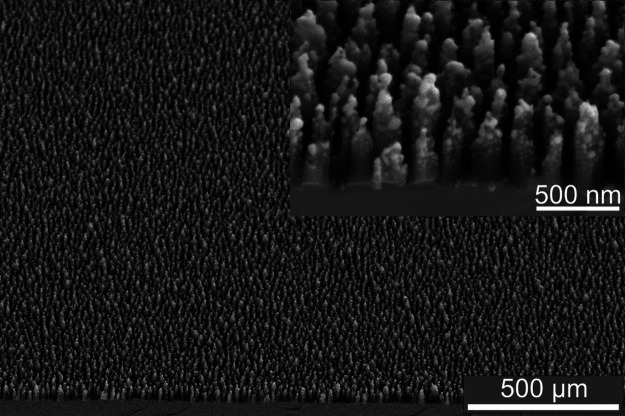
Electron images of the AuBSi nanostructure. Images (collected at 45°) show the nanostructure: the silicon nanopillars (in dark gray) and the gold nanoparticles (in bright gray or white) create a nanoasparagus architecture, better visualized in the inset.

The gold-coated black silicon (AuBSi)-nanostructured substrate used in this work was previously developed by our group for surface-assisted laser desorption/ionization mass spectrometry imaging applications.^22^ Briefly, the substrate is a silicon wafer with a homogeneous-nanostructured surface, which consists of ∼500 nm tall silicon nanopillars decorated with 10–20 nm gold nanoparticles, resembling a forest of nanoasparagus (Figure 1). The roughness of the nanostructure is ideal for capturing a high number of molecules from liquid or solid samples due to the high surface area of the nanopillars (Figure S1A). The substrate’s surface chemistry is stable over more than 17 months, ensuring its correct functionality after storage in normal conditions (room temperature and atmospheric pressure; Figure S1B). Lastly, the substrate absorbs >90% of light, including the wavelengths of the lasers used in this study: 355 nm for SALDI and 514, 633, and 785 nm for Raman (Figure S1C).

#### AuBSi for SALDI MSI

We tested the potential of the AuBSi substrate for SALDI measurements by comparing the spectra collected from rhodamine 6G (R6G) spotted on a bare silicon wafer and our substrate. Figure S2 illustrates that even self-assisting compounds such as dyes cannot be detected with low laser fluency (with our MADLI instrument) and that our substrate helps ionizing the molecule without generating fragments. Then, we demonstrated the use of the AuBSi substrate in MSI. Aqueous solutions (1 mM) of R6G and MG were spotted with an inkjet printer on the substrate (Figure S3) for testing the optimal laser desorption/ionization (LDI) performance. Figure S4A shows the average spectra of an area collected in the negative polarity mode with the most intense peak at *m*/*z* 96 putatively associated with a fragment of the molecules or background signal and the typical peaks of Au (*m*/*z* 196) and its clusters (*m*/*z* 393, 590, 787, and 984). In this case, the signal from rhodamine 6G and MG cannot be detected, as they are cationic basic dyes that do not ionize in the negative mode. Figure S4B shows the average spectra of the inkjet-printed R6G and MG solutions in the positive ionization mode. The most intense peaks at *m*/*z* 443 and *m*/*z* 329 come from rhodamine 6G ([M + H–2H_2_O]^+^ adduct) and MG ([M + H–2H_2_O]^+^ adduct), respectively. No signal interference from the background or the Au nanoparticles was observed.

#### AuBSi for SERS Imaging

The characteristics of the AuBSi substrate make it also optimal for SERS measurements. First, we tested which laser line works best with the AuBSi substrate by mapping the printed R6G and MG droplets with three different laser wavelengths: 514, 633, and 785 nm. The single spectra of R6G and MG from each droplet collected with each laser, together with images representing the intensity distribution of the specific bands of R6G and MG are illustrated in Figure S5. The best quality spectra were collected with the 633 nm laser, showing the highest intensity and the best definition between different bands (Figure S5A). We chose to represent R6G with the band 1513 cm^–1^ (characteristic for C–C stretching)^37^ and MG with the band 1620 cm^–1^ (characteristic of the benzene ring C–C stretching)^38^ as they were the most intense bands characteristic to each probe. AuBSi with the 633 nm laser works best for detecting both R6G and MG due to the following: (1) effective adsorption of molecules to the surface, (2) high absorption of the laser energy by the substrate, and (3) the presence of Au nanoparticles which lead to enhanced electromagnetic fields.^39,40^

**Figure 2:**
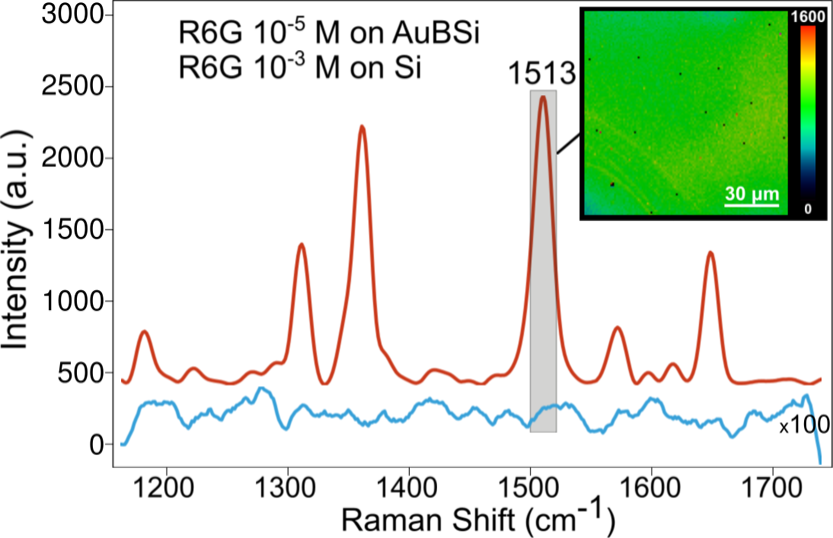
Characteristics of the AuBSi substrate for SERS imaging measurements. Mean spectrum of 50 × 50 μm^2^ area mapped on the Si (in blue) and AuBSi (in red) substrates immersed in 10^–3^ and 10^–5^ M rhodamine 6G solution, respectively; the spatial distribution of the 1510 cm^–1^ band at 30 μm spatial resolution of R6G is represented in the inset (arbitrary intensity units, 0 to 1600). Average spectrum collected from Si (in blue) is multiplied 100 times for the purpose of comparison. Black pixels are excluded spectra during pre-processing.

Then, we characterized the substrate homogeneity, a paramount property for imaging experiments. For this, we mapped the AuBSi surface after R6G immersion. Figure 2 shows the mean spectra of the map collected and the distributions of the most intense band of R6G. The bands at 1315, 1360, and 1513 cm^–1^ are associated with aromatic C–C stretching vibrations. The map shows a highly uniform distribution of the SERS intensity at 1510 cm^–1^ when scanning with 1 μm lateral resolution, that is, without any significant hot-spots that could mislead qualitative or quantitative analyses. Therefore, the AuBSi substrates can be used for imaging fine morphological and chemical details. Next, we calculated the analytical enhancement factor (AEF) of the substrate^39^ (explained in the Supporting Information file—page S-6). As controls, we measured the normal Raman spectrum for R6G by immersing the bare silicon (Si) and black silicon (BSi) substrates in aqueous R6G solution (10^–3^ M) for 1 h, and the SERS spectra by immersing the AuBSi substrate in aqueous R6G solution (10^–5^ M) for 1 h. To avoid measuring molecules not directly attached to the surface, each substrate was rinsed by dipping in distilled water three times. The obtained AEFs for the AuBSi substrate were 5.4 × 10^5^ when comparing to Si and 2.3 × 10^5^ when comparing to BSi (see Figure 2). We finally tested the linearity of the AuBSi substrate, as SERS spectroscopy can also be used for quantitative studies.^15,37^ In our case, the intensity of the band 1513 cm^–1^ was linearly correlated to the R6G concentration between the ranges of 1 μM and 1 mM (Figure S6).

#### AuBSi for Multimodal Imaging

To perform SALDI and SERS imaging on the same sample, we first determined the optimal acquisition order. We acquired maps of AuBSi immersed in the R6G solution first by SALDI and then by SERS and vice versa (Figure S7). The SALDI spectra were not negatively affected by the SERS measurement, but the SERS spectra intensity decreased after the SALDI measurement (this was expected, as MS techniques are destructive). This behavior was maintained even when the SERS map was acquired at a 10-fold higher resolution (2 μm as spot spacing, see Figure S8). Even though we could not detect any serious sample deterioration from the Raman spectra, or substrate damage through optical images (Figure S9), we consider that it is best to measure with SERS first and with SALDI last to retain the best signal intensity.

**Figure 3:**
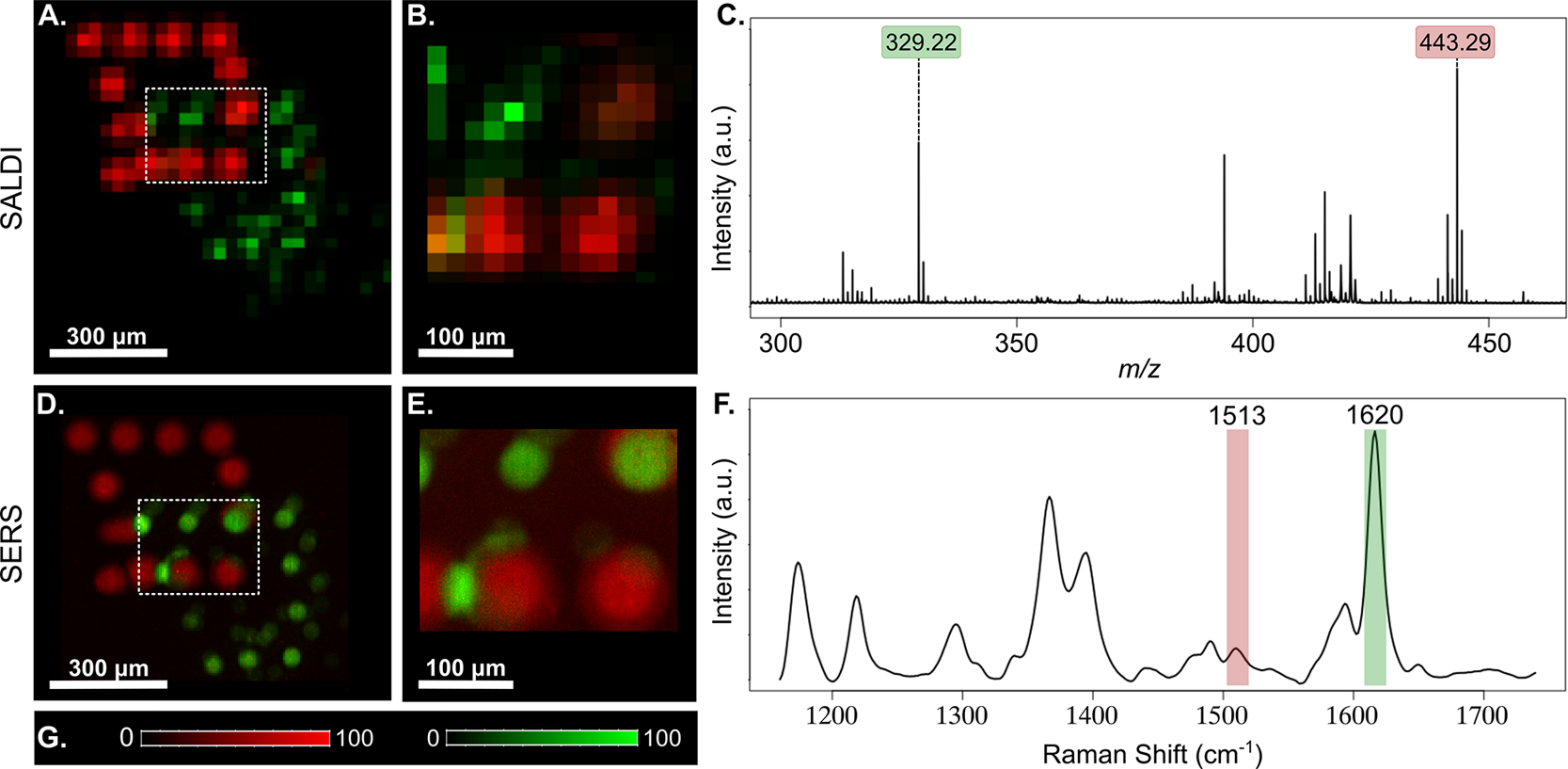
SALDI and SERS images and their average spectra of rhodamine 6G and MG. (A) 30 μm resolution and (B) 20 μm resolution SALDI ion maps represented by *m*/*z* 329.22 in green and *m*/*z* 443.29 in red detected as [M + H – 2H_2_O]^+^ adducts. (B) Covers the dashed area in (A). (C) Average mass spectrum of (B) highlighting the represented ions. (D) 5 μm resolution and (E) 1 μm resolution SERS maps; 1513 cm^–1^ band (in red) is the C–C stretching vibration of rhodamine 6G and 1620 cm^–1^ band (in green) is the benzene ring C–C stretching vibration of MG. (E) Covers the dashed area in (D). (F) Average SERS spectrum of the 1 μm map highlighting the represented bands. (G) Intensity scales for the red and green channels.

For comparing the lateral resolution capabilities of SERS and SALDI, R6G, and MG microliter droplets were spotted with an inkjet printer in the shape of two overlapping squares (represented in Figure S3). The slightly skewed shape of the squares and the letters is the result of imperfect printing conditions; however, the shapes and letters can be distinguished even after 12 layers of printing. The intensity maps of the characteristic ion peaks and Raman bands are illustrated in Figure 3. For the SALDI-MS image, the best lateral resolution we could achieve was 20 μm (apparatus limitation), while for the SERS image, we acquired at 1 μm lateral resolution. Figure 3B,E shows the regions of SALDI and SERS images, respectively, where the R6G and MG droplets are overlapping, displaying the poorer lateral resolution capabilities of SALDI-MSI.

#### Image Coregistration Strategy

Coregistration allows us to integrate data from multiple imaging modalities by aligning them in space.^41,42^ The simple and distinct morphologies of the ink droplets were used to optimize and validate our coregistration strategy (as illustrated in Figure S10) which consists of four steps: (1) selecting morphologically representative images; (2) revising resolution discrepancy; (3) using teaching points to create a transformation matrix; and (4) running the automatic coregistration. This coregistration approach works correctly despite the very different lateral resolutions of the two images. Thus, coregisteration generates a coordinate map which can be used to retrieve SERS and SALDI-MS spectra for the same location. This will enable straightforward multimodal data analysis.

### Multimodal Imaging Detects Residues from Fingermarks

We developed a multimodal imaging approach with the help of the AuBSi substrate to analyze the molecules transferred onto the substrate surface from biological samples. We tested our approach by analyzing endogenous and exogenous molecules from fingermarks. The workflow consists of five steps: (1) molecule transfer (i.e., stamping the fingermark); (2) image acquisitions (first SERS then SALDI); (3) separate data pre-processing; (4) image coregistration; and (5) data analysis. The aim of this workflow is to obtain rich localized information which can discriminate between clean and stained fingermarks and provide chemical information about the staining compounds.

**Figure 4:**
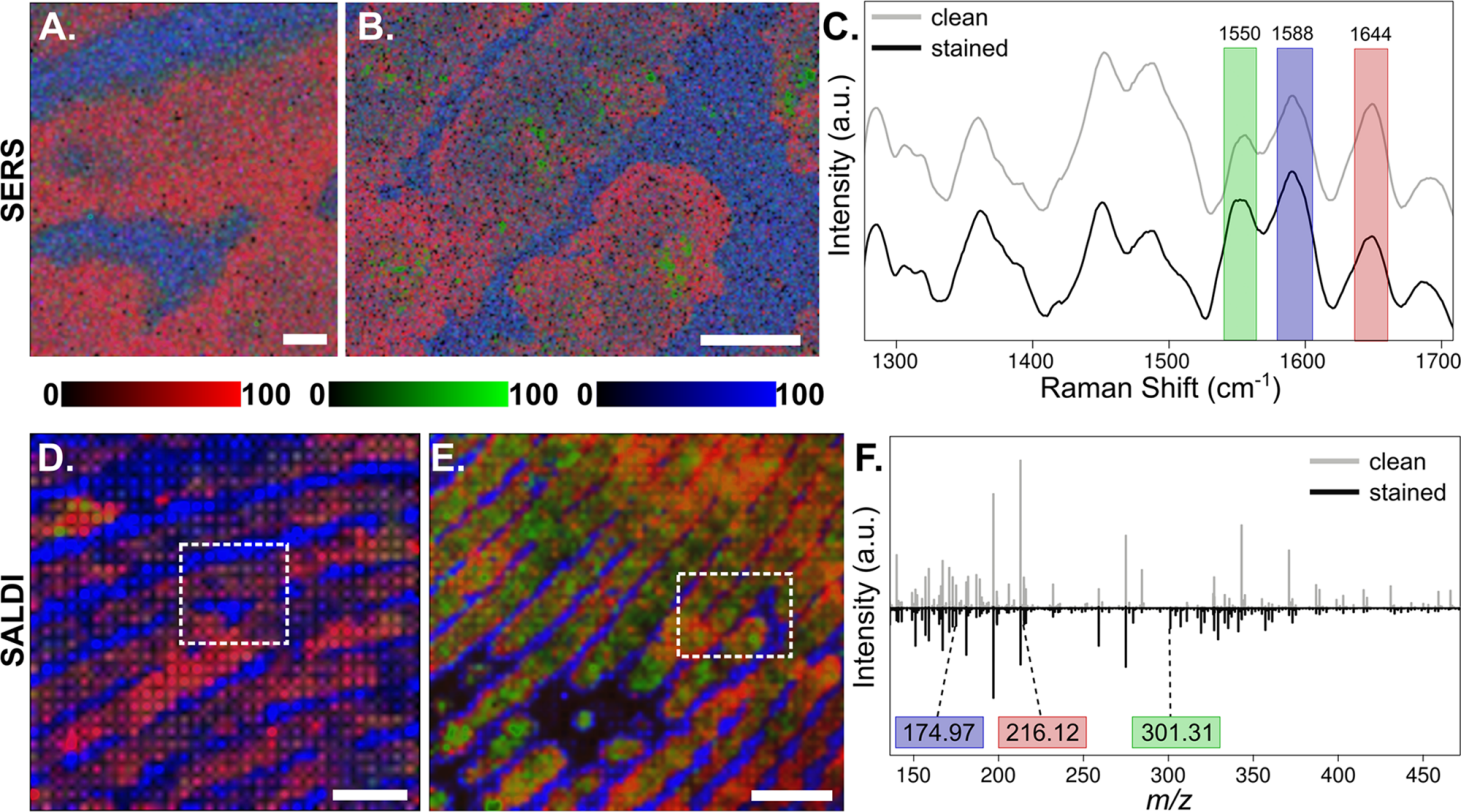
SERS and SALDI measurements of clean and stained fingermarks. (A) 5 μm resolution and (B) 7 μm resolution RGB channel band maps for clean and stained fingermarks, respectively; (C) average SERS spectra representing the bands 1644 cm^–1^ in red (ridge), 1588 cm^–1^ in green (stain), and 1550 cm^–1^ in blue (valley); 50 μm resolution RGB channel ion map for (D) clean and (E) stained fingermarks; (F) average mass spectra from (D,E), highlighting the ions *m*/*z* 216.12 in red (ridge), *m*/*z* 301.31 in green (stain), and *m*/*z* 174.97 in blue (valley); dashed squares mark the SERS area measurement in (A,B), respectively. Scale bars: 100 (A), 200 (B), 600 (D), and 800 μm (E).

SERS images were collected at high resolutions (5 and 7 μm) to study the morphological details of a clean fingermark and a fingermark stamped after rubbing the finger with plasticine modeling clay (clean and stained fingermarks, respectively). Although Raman maps are generally acquired at ∼1 μm resolution, we chose to collect maps at 5 and 7 μm for three reasons: (1) the spatial resolution is enough to reflect the morphology of fingermarks, as the sweat and eccrine glands pore size can be distinguished at this resolution;^43^ (2) SERS and SALDI image coregistration is favored when the spatial resolution discrepancy is not too high;^27^ and (3) these parameters ensure the reduced acquisition time (at this resolution, due to the large size of the images: 0.8 × 0.7 mm^2^ for the clean and 0.75 × 1 mm^2^ for the stained fingermark; the acquisition time was ∼7.5 and ∼12 h, respectively). Figure 4 illustrates the distribution of skin-related compounds (represented by the bands at 1588 cm^–1^ in blue for the valley and 1644 cm^–1^ in red for the ridge) and of plasticine molecules (tentatively associated with the 1550 cm^–1^ band in green for the stain). The morphology of the fingermark can be easily distinguished as the 1588 cm^–1^ band (tentatively associated with amino acids—Tyr, Phe, Trp—due to the aromatic ring breathing^44^) is representative of the valley and the 1644 cm^–1^ band (associated with proteins due to the amide I vibrations^45,46^) is representative of the ridge. The average spectrum of the clean (in gray) and stained (in black) fingerprint maps show the typical bands and shoulder bands of biomolecules found in the fingermark composition, while the reference spectrum collected from plasticine (Figure S11) shows different bands specific to the molecules composing the plasticine modeling clay. All putatively identified bands and shoulder bands are listed in Table S2.

SALDI-MS images were collected from the clean and stained fingerprint residue transferred onto the AuBSi surface at a 50 μm lateral resolution. We detected a wide range of *m*/*z* signals potentially associated with molecules not only originating from the sebaceous and eccrine glands but also from contaminants such as dust, cosmetics, food residue, plastics, and their metabolites.^47^ As shown in Figure S12, these molecules could not be ionized and detected from fingerprints stamped on bare silicon at any laser fluency. Figure S13 illustrates the average spectrum of the clean and stained fingermark maps on AuBSi, together with the reference spectrum collected from the plasticine. We detected several morphologically relevant *m*/*z* features: exogenous from the plasticine and endogenous from the finger sweat (e.g., likely lipids, proteins, amino acids, fatty acids, wax esters, and so forth of the eccrine and sebaceous origin)^47,48^ (Table S3).

#### Multimodal Approach

**Figure 5:**
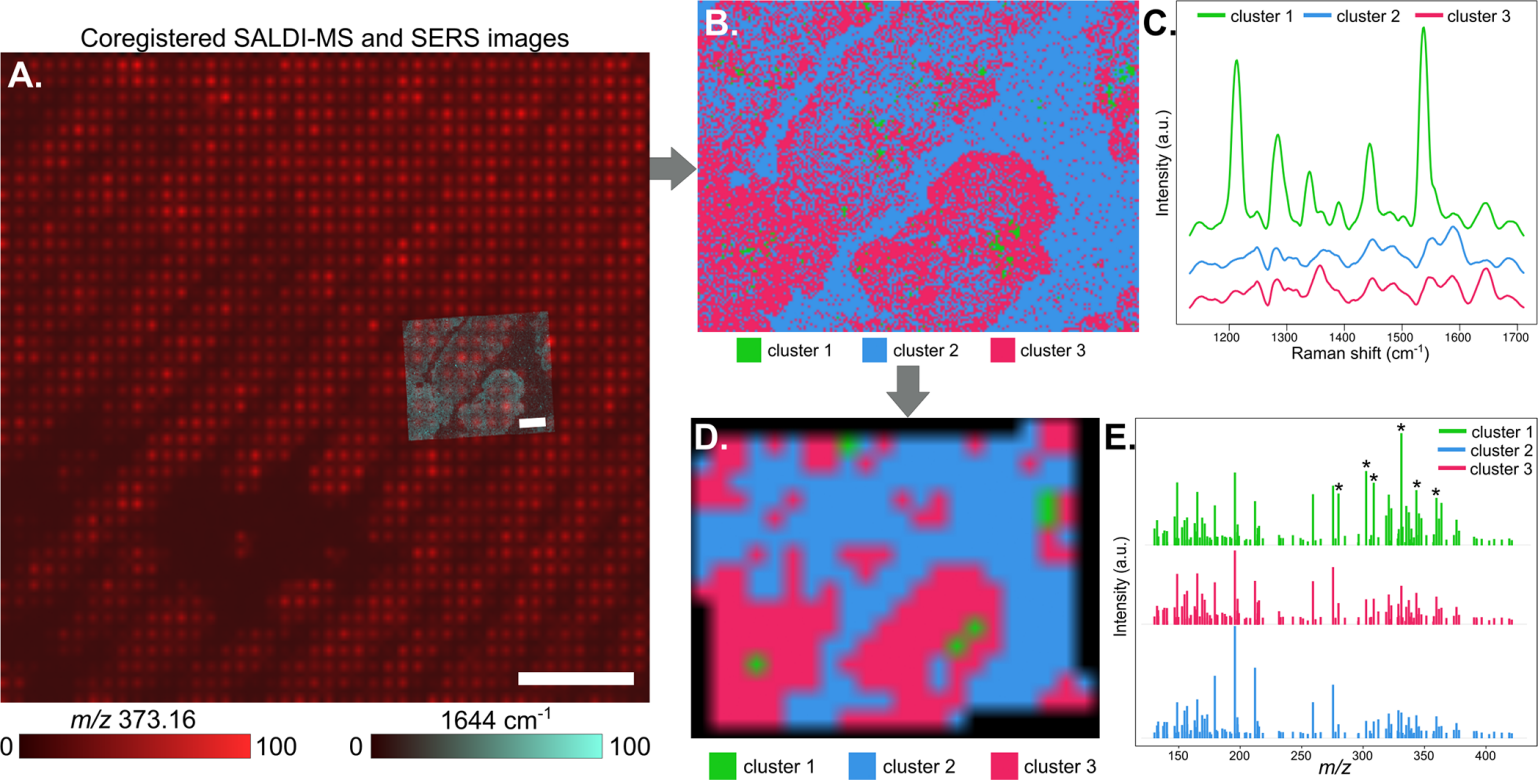
Multimodal analysis strategy. (A) Coregistered SALDI-MS (in red ions *m*/*z* 373.26) and SERS (in the blue band 1644 cm^–1^) images; (B) SERS *k*-means cluster map; (C) associated average spectra; (D) SERS clusters retrieved from coregistration and assigned to the SALDI-MS pixels; and (E) the average mass spectra of each transformed cluster. Asterisks mark the peaks present only in cluster 1, which were assigned to the signal from plasticine; scale bar for the SALDI ion image (in red) is 800 μm and for the SERS band image (in blue) is 200 μm.

Given that the staining molecules only cover a fraction of the acquired area, identifying them through their corresponding spectral features (SERS bands and SALDI *m*/*z* peaks) from the mean spectra is unfeasible. Additionally, the lateral resolution of SALDI is often not enough to detect the small regions of staining molecules. To overcome these limitations, we coregistered the SERS and SALDI images. First, we aligned the two experiments using our in-house algorithm relying on manual teaching points. Then, we automatically segmented the SERS image using *k*-means clustering. The resulting segmentation was then used to retrieve the SALDI-MS pixels and assign their respective cluster, as illustrated in Figure 5. The average spectra of each cluster from the SERS image reveal bands that were not distinguished in the mean spectrum of the whole image (Figure 4C). Specifically, cluster 1 in Figure 5B seems to represent the staining molecules from the fingermark, as its spatial pattern is spotty, and its spectrum is very distinct from the fingermark composition from the other two clusters (Figure 5C) and from the average spectrum of the image (Figure 4C). The corresponding SALDI cluster 1 (Figure 5D) also presents prominent *m*/*z* features not present in other clusters, marked with a star in Figure 5E. The most intense pixels of these ion images (as reflected in the ion images from Figure S14) coincide with the position of the SALDI cluster 1 in Figure 5C.

The label that the manufacturer includes in the package of the plasticine modeling clay describes the generic composition of the clay: wax, starch, white oil, and pigments. Several ions specifically concentrated in cluster 1 were putatively identified as fatty acids, esters of fatty acids, or aliphatic compounds [e.g., *m*/*z* 305.34 [M + Na]^+^ of C_18_H_34_O_2_ oleic acid; *m*/*z* 307.35 [M + H–H_2_O]^+^ of C_21_H_39_O wax ester WE(21:1); *m*/*z* 327.30 [M + H]^+^ of C_21_H_43_O_2_ wax ester WE(21:0)],^49^ consistent with the information from the label. On the other hand, the SERS average spectra of cluster 1 has its maximum band at 1540 cm^–1^, common in Cu-phthalocyanine dyes,^50^ due to the stretching of the C–N bonds in the macrocycle. Thus, the green pigment in the plasticine is most likely Pigment Green 7 (PG7).^51^ To corroborate these findings, we analyzed by SALDI and SERS three samples of plasticine of three different colors: white, yellow, and green (the latter was the same plasticine used in the fingermarks experiments). We found that all plasticine samples contained the ions between *m*/*z* 279 and *m*/*z* 327 that we putatively identified as hydrocarbons, thus these ions are most likely associated with the wax and white oil (Figure S13). However, the band at 1540 cm^–1^ in the SERS spectra was only present in the green plasticine but not in the white or yellow samples; therefore, this band is definitively linked to the green pigment PG7 (Figure S11).

Our multimodal imaging approach uses an AuBSi substrate to combine SALDI-MS and SERS imaging with simple sample preparation suitable for both techniques. The AuBSi substrate is ideal for a cost-effective and user-friendly substrate for molecule detection using laser-based imaging techniques. Moreover, the gold nanoparticles decorating the substrate are ideal enhancing agents for both SALDI and SERS, as they ensure background-free and enhanced signals, and promote measurements with the highest lateral resolution. AuBSi performed best in the positive mode in SALDI measurements (due to the nature of the Raman reporters) and worked best with the 633 nm laser with SERS (due to the resonance of the Au nanoparticles). The effectiveness of the substrate for SERS imaging was evidenced by its AEF (in the order of 10^5^); its ability to detect molecules from solutions or from solid samples; and its homogeneous surface which allows SERS mapping at micron-scale resolution. Using orthogonal analytical techniques to measure the same sample, on the same substrate with the same sample preparation, promotes synergistic analysis: the limitations of one technique can be addressed by the advantages of the other. We combined SALDI MSI and SERS to achieve three main goals:

- *Easy sample preparation.* Sample preparation for multimodal imaging using the AuBSi substrate is straightforward because it only consists in depositing the target analytes onto the substrate. After this, measurements can be done either in atmospheric conditions or in vacuum, depending on the technique. Printing Raman reporter inks, immersion in Raman reporter solutions or even touching the finger to the surface of the substrate are convenient sample preparation protocols that can be reproduced easily. It is noteworthy that the AuBSi substrate also supports non-imaging applications. Moreover, SERS and SALDI measurements can be done in any order, demonstrating the flexibility of AuBSi to be used for the multimodal imaging of all kinds of samples.
- *Improved lateral resolution.* SERS maps can provide high-resolution images which can be spatially analyzed with multivariate tools such as *k*-means clustering, to define regions of interest in otherwise overlooked areas in MSI studies. Fingermark morphology can be easily distinguished by both SALDI and SERS univariate analyses, but the presence of staining molecules cannot be confidently represented without image coregistration and multimodal data analysis. For example, the plasticine contaminants were considerably lower in size than the SALDI pixel (<20% of their area). Because of the coregistration of the two modalities, we were able to label the stained SALDI pixels that overlapped with at least 15% of stained SERS pixels. Coregistration with SERS allowed us to overcome the intrinsic resolution limitation of SALDI and detect contaminants smaller than its pixel size.
- *Complementary molecular information.* SERS and SALDI are orthogonal analytical techniques that provide complementary molecular information. SERS is a powerful spectroscopic technique which informs on the vibrational structure of molecules, while SALDI gives precise information about the mass-to-charge ratio of monomers, isotopes, adducts, and fragments. In targeted studies—such as the experiment from Figure 3—identifying molecules is straightforward in both SALDI and SERS, but for untargeted studies identifying biomolecules with a single technique is challenging. In these cases, having complementary molecular information is very valuable. In our study, the staining molecules on the fingermark were detected by *k*-means clustering on the two spatially correlated datasets. Specifically, the multimodal approach linked SERS and SALDI pixels and their respective spectral data, which enabled identifying features such as the bands 1215 and 1540 cm^–1^ and ions *m*/*z* 301.31 and *m*/*z* 307.35 associated with the staining molecules from the plasticine.

Still, not all challenges of the two modalities can be overcome by multimodal imaging. For example, our substrate enhances the Raman signal of the molecules in the close vicinity to the AuNP, so SERS imaging of thicker samples, such as tissues—which have far greater amounts of molecules residing outside the enhancing electromagnetic field—is troublesome. In SALDI, a similar challenge appeared: the laser could not interact with the AuNP due to the thickness of the tissue sample, which resulted in poor ionization yield. Our solution for SALDI-MSI was to remove the excess material by washing,^22^ which concentrated the analysis exclusively on the molecules adhered to the surface of the nanostructure. This approach was successful for investigating fingermarks but similarly prepared samples, such as imprinted molecules from plants or even liquid samples can be analyzed.

Our AuBSi substrate works as a sample support which enhances the Raman signal in SERS imaging and promotes the ionization of molecules in SALDI-MSI, independently of the method. The possibility to measure from the same sample and to coregister the acquired images allow high confidence identification of molecules based on multivariate data analysis.

## Conclusions

Our multimodal imaging approach uses the same substrate, the same sample, the same sample preparation method, and the same data analysis procedures. We mapped the molecular composition of fingermark residues at high lateral resolution using SERS and detected endogenous and exogenous compounds using SALDI. This approach can be translated into fields such as clinical, environmental, forensics, and pharmaceutical research, where Raman spectroscopy is an established technique.

## Supporting Information Available

Comparison between SERS imaging, SALDI MSI, and multimodal imaging on AuBSi; physico-chemical characterization of the AuBSi substrate; AuBSi performance for SALDI-MSI and SERS imaging; coregistration strategy; and tentative annotation of bands (SERS) and ions (SALDI) detected from the same fingerprint sweat on AuBSi (PDF)
