# Supplementary material for: SALDI-MS and SERS Multimodal Imaging: One Nanostructured Substrate to Rule Them Both
Source: Anal Chem. 2022 Feb 1;94(6):2785–93. doi: 10.1021/acs.analchem.1c04118 (PMC8851428; doi:10.1021/acs.analchem.1c04118)
Supplement: Supplementary file 1 — ac1c04118_si_001.pdf [file ac1c04118_si_001.pdf]

Supporting Information for SALDI-MS and SERS Multimodal Imaging: one nanostructured substrate to rule them both

Stefania-Alexandra Iakab[1,2,*] , Gerard Baquer[1], Marta Lafuente[3,4], Maria Pilar Pina[3,4,5], José Luis Ramírez[1], Pere Ràfols[1,2], Xavier Correig-Blanchar[1,2,6], María García-Altares[1,2]

[1]Department of Electronic Engineering, Rovira i Virgili University, Tarragona, 43007, Spain

[2]Spanish Biomedical Research Centre in Diabetes and Associated Metabolic Disorders (CIBERDEM), Madrid, 28029, Spain

[3]Instituto de Nanociencia y Materiales de Aragón (INMA), CSIC-Universidad de Zaragoza, 50009, Zaragoza, Spain.

[4]Departamento de Ingeniería Química y Tecnologías del Medio Ambiente, Universidad de Zaragoza, Campus Río Ebro-Edificio I+D+i, C/Mariano Esquillor s/n, 50018, Zaragoza, Spain.

[5]Networking Research Center on Bioengineering, Biomaterials and Nanomedicine, CIBER-BBN, 28029, Madrid, Spain.

[6]Institut d'Investigació Sanitària Pere Virgili (IISPV), 43204 Reus, Spain

*Corresponding authors: s.iakab@hs-mannheim.de and maria.garcia-altares@urv.cat

Table of Contents:

Comparison between SERS imaging, SALDI MSI and multimodal imaging on AuBSi; physico-chemical characterization of the AuBSi substrate; AuBSi performance for SALDI-MSI and SERS imaging; coregistration strategy; tentative annotation of bands (SERS) and ions (SALDI) detected from the same fingerprint sweat on the AuBSi.

Table S1. Comparison between standard SERS and SALDI imaging and our approach to multimodal imaging on AuBSi substrate.

**Technique/ Aspect**	**SERS**	**SALDI**	**Multimodal imaging on AuBSi**
**Substrate**	Low fluorescence CaF_2 substrate	ITO-covered glass slide	AuBSi multimodal substrate
**Sample Preparation**	• Tissue mounted on CaF_2 substrate + nanoparticle deposition<br>• Tissue incubated with nanostructured material	• Tissue mounted on ITO-covered glass slide + nanoparticle deposition<br>• Tissue mounted on nanostructured substrate	• Tissue mounted on AuBSi substrate followed by tissue removal for molecular transfer
**Spatial Resolution**	<1µm	>20µm	Both
**Spectrum Quality**	low	high	Multimodal data
**Sensitivity**	low	high	Multimodal data
**Specificity**	medium	high	Multimodal data
**Acquisition Time**	Medium to long: Parameter and instrument dependent	Long: Parameter and instrument dependent	Depends on acquisition technique/method
**Data Format**	Instrument specific formats; Exported .txt files	imzML	imzML*
**Data Processing**	Baseline Correction<br>Cosmic Ray Removal<br>Smoothing<br>Normalization	Alignment<br>Smoothing<br>Peak Picking<br>Normalization	All
**Type of Experiment**	Targeted<br>Analysis<br>(not metabolomics)	Targeted<br>Untargeted<br>Analysis	All
**Chemical Information**	Functional Groups<br>Chemical Bonds<br>Molecular Conformation	Molecular Weight (and ion fragmentation patterns)<br>Isotopic Patterns<br>Adduct Formation	All

*SERS imaging data is converted into imzML using Raman2imzML converter

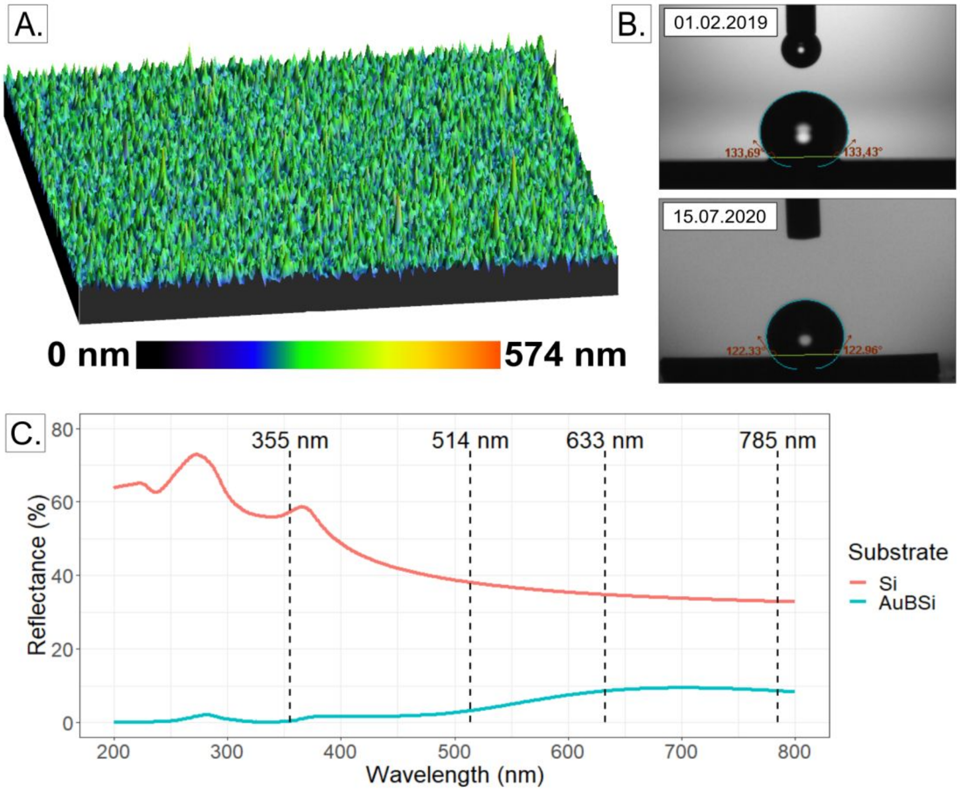

Figure S1. AuBSi surface properties. A: AFM roughness map of the AuBSi surface; **B:** Contact angle measurements with water on the AuBSi over a period of 17 months; and **C:** Reflectance measurements comparing normal Si wafer and AuBSi behavior when interacting with the lasers used in MSI (355 nm) and Raman (514 nm, 633 nm, 785 nm) measurements.

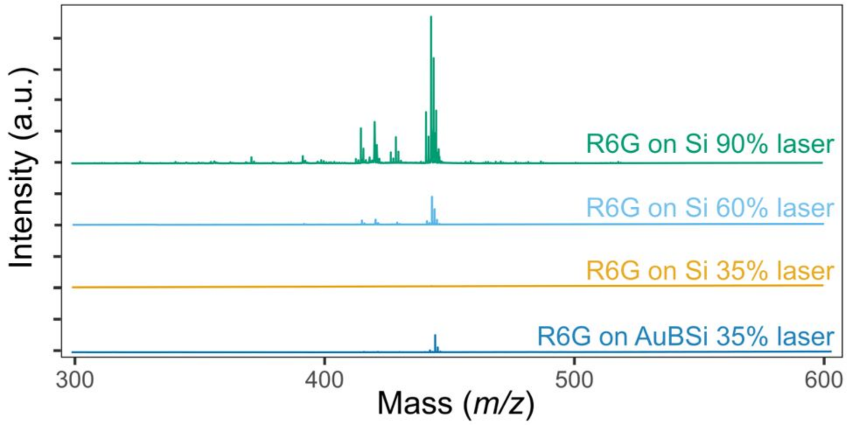

Figure S2. Comparison of R6G spectra (1mM solution) collected from a bare Si wafer and our AuBSi at three different laser power settings (35%, 60%, 90%).

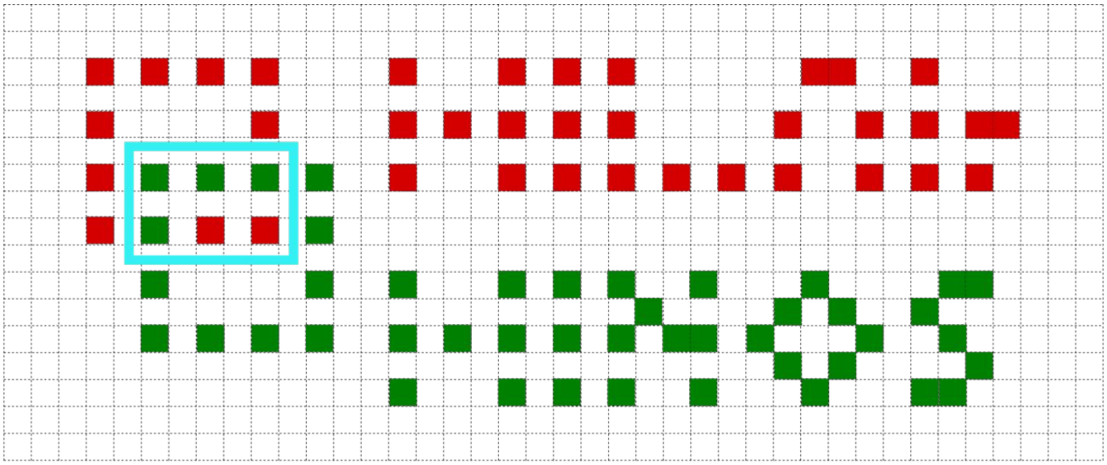

Figure S3. Reference layout used for Inkjet printing. Two overlapping squares and the names of two research groups from the department where the research was carried out. The ink used for the red spots contains rhodamine 6G (R6G) and the one used for the green spots contains malachite green (MG); droplet diameter was between 90-100 µm and between 60-80 µm, respectively. The blue rectangle marks the region measured in Figures 3, S4 and S10. The printing method can be found in the methods section in the main manuscript.

Inkjet printing technical difficulties: The layout from Figure S2 was not perfectly printed onto the surface of the AuBSi substrate, as the resulting droplet position was slightly skewed to the right. Nevertheless, the shapes and letters could be distinguished even after 12 layers of printing.

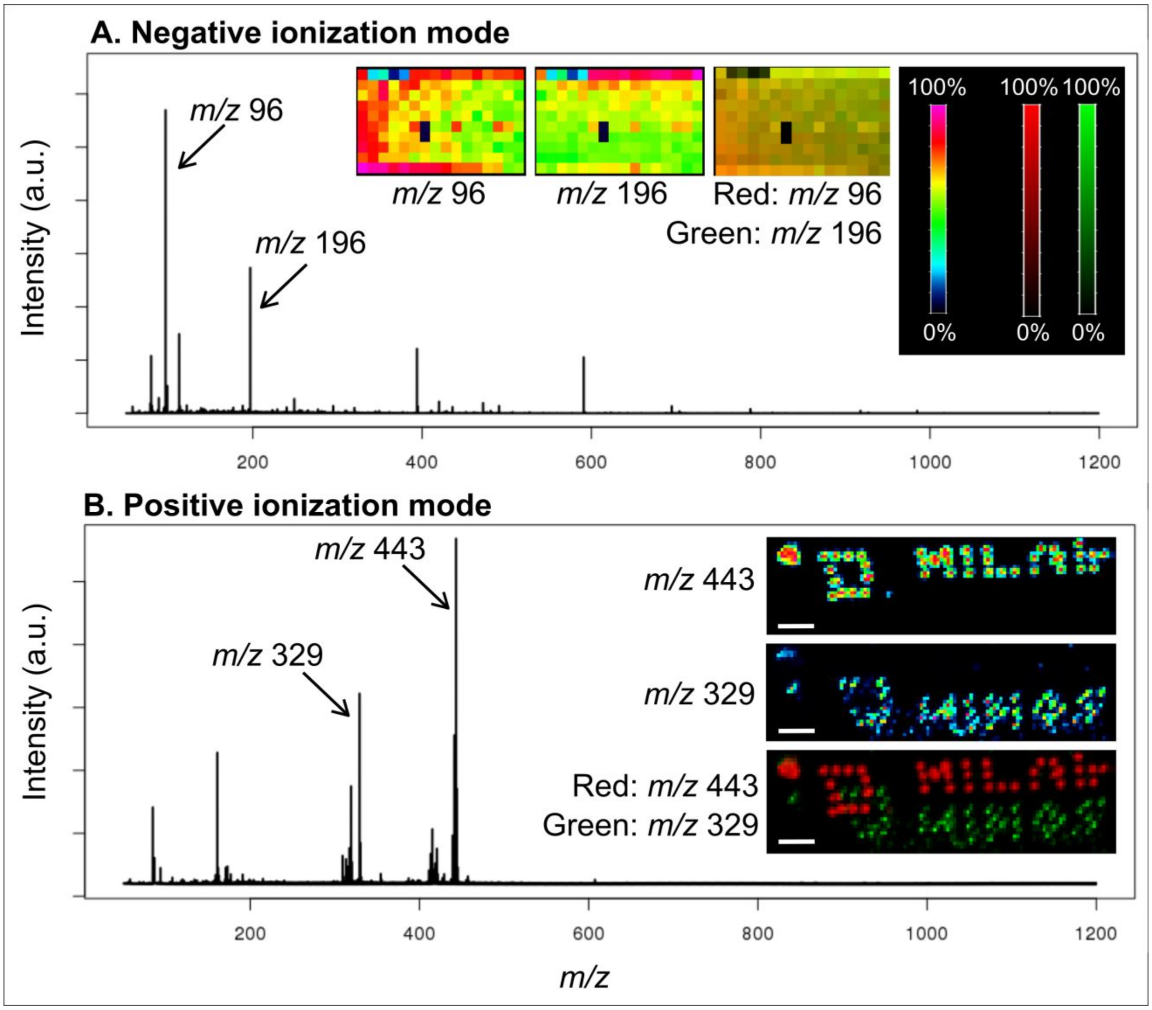

Figure S4. Evaluating negative and positive ionization modes of R6G and MG inks printed on AuBSi. A: Average spectra and ion intensity distribution images of the first two most intense peaks: *m/z* 96 from background and *m/z* 196 from Au^- ions in negative ionization mode; area measured 4 x 2.5 mm^2 at 250 µm lateral resolution. **B:** Average spectra and ion images (lateral resolution) of rhodamine 6G (in red, *m/z* 443.29) and malachite green (in green, *m/z* 329.22) ions detected as $[M+H-2H_2O]^+$ adducts, printed on the AuBSi substrate as the acronyms of two research groups (MILAB and MINOS - represented in Figure S2) from our department; scale bar is 500 µm; area measured 3.1 x 1 mm^2 at 30 µm lateral resolution.

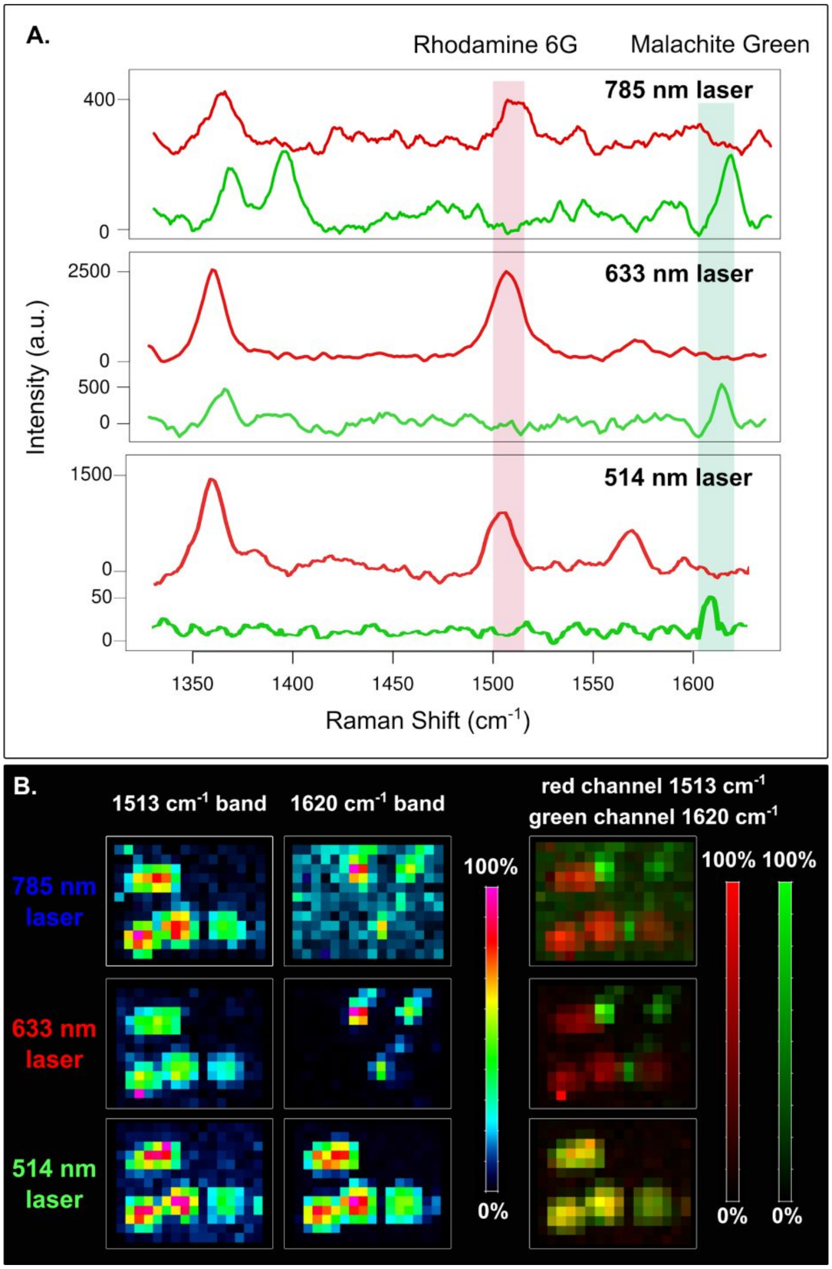

Figure S5. AuBSi compatibility with lasers from the visible range. A: Single pixel spectra from the rhodamine 6G droplet (in red) and from the malachite green droplet (in green) with three lasers: 514 nm, 633 nm and 785 nm wavelength; bands specific to Rhodamine 6G (1513 cm^{-1}) and malachite green (1620 cm^{-1}) are highlighted with transparent red and green, respectively. **B:** Raman images collected with the three lasers: individual and two channel representations of the distribution of the two bands 1513 cm^{-1} and 1620 cm^{-1} and for each laser; measurement area 0.32 x 0.24 mm^2 at 20 µm lateral resolution. The acquired measurement area is marked with a blue rectangle in Figure S2.

Calculating the analytical enhancement factor (AEF):

The AEF quantifies the increase of signal intensity compared to normal Raman spectroscopy under specific experimental conditions and is calculated using the following equation:

$$\mathrm{AEF} = \frac{I_{\mathrm{SERS}}/(t_{\mathrm{SERS}} \times P_{\mathrm{SERS}} \times C_{\mathrm{SERS}})}{I_{\mathrm{Raman}}/(t_{\mathrm{Raman}} \times P_{\mathrm{Raman}} \times C_{\mathrm{Raman}})}$$

where C_{Raman} and C_{SERS} are the R6G concentration in the Raman (1 mM) and SERS (1 μM) measurement conditions, respectively and I_{Raman} and I_{SERS} are the intensity values of the R6G characteristic band at 1510 cm^{-1}, for the normal Raman and SERS measurements, respectively[1]. Similarly, P_{Raman} and t_{Raman} and P_{SERS} and t_{SERS} refer to the laser power and acquisition time values for the normal Raman and SERS measurements, respectively. In our case, laser power and exposure time were the same, but the normal Raman R6G spectrum was measured immersing a bare silicon wafer or BSi substrate in 10^{-3} M R6G solution and the AuBSi substrate in 10^{-5} M R6G solution for 1 h then rinsed with distilled water by dipping 3 times to remove excess molecules not adhered to the surface. Thus, I_{SERS} is the intensity of the R6G molecules that remain adsorbed on the SERS substrate and I_{Raman} is the intensity of the R6G molecules adsorbed on the bare Si. Based on this calculation approach, the AuBSi substrate has an AEF of $2.3 \cdot 10^5$ when compared to BSi and $5.4 \cdot 10^5$ when compared to Si. Similarly, when using the most intense band from malachite green for the calculation, the AEF of AuBSi is $7.5 \cdot 10^3$ when compared to Si and $2.3 \cdot 10^3$ when compared to BSi.

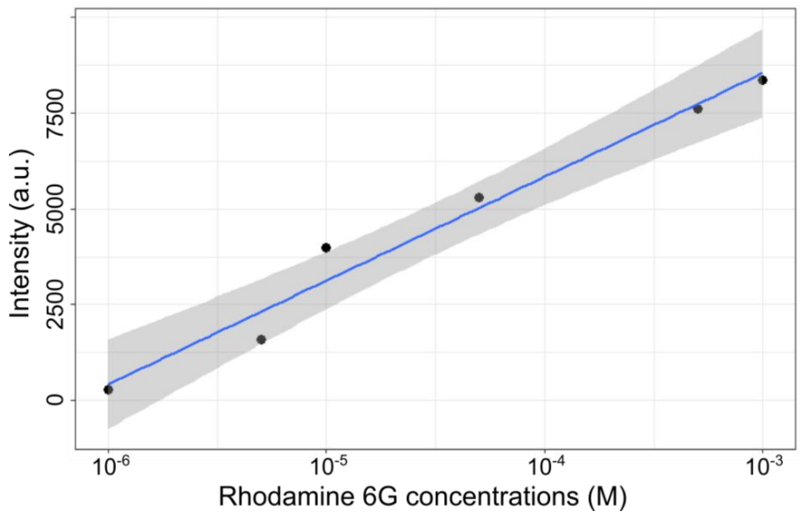

Figure S6. Substrate linearity. Regression line obtained from the average intensity of the 1510 cm^{-1} band from 100 pixels with different R6G concentrations. The standard deviation of the mean is highlighted in grey relative to the blue line fit.

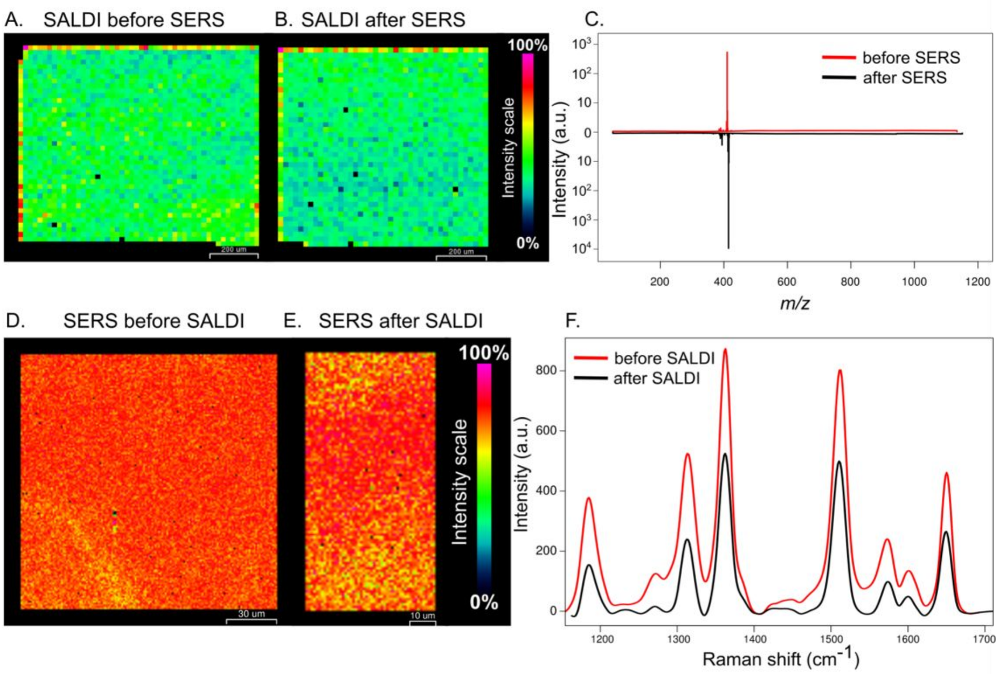

Figure S7. Imaging acquisition order assessment. A: ion map (*m/z* 443) collected before SERS measurement. **B:** ion map (*m/z* 443) collected after SERS measurements. **C**: average spectrum of the maps from (A) in red and (B) in black. **D**: band map (1362 cm^{-1}) collected before SALDI measurement. **E**: band map (1362 cm^{-1}) collected after SALDI measurement. **F**: average spectrum of the maps from (D) in red and (E) in black. All maps were acquired at 20 µm lateral resolution.

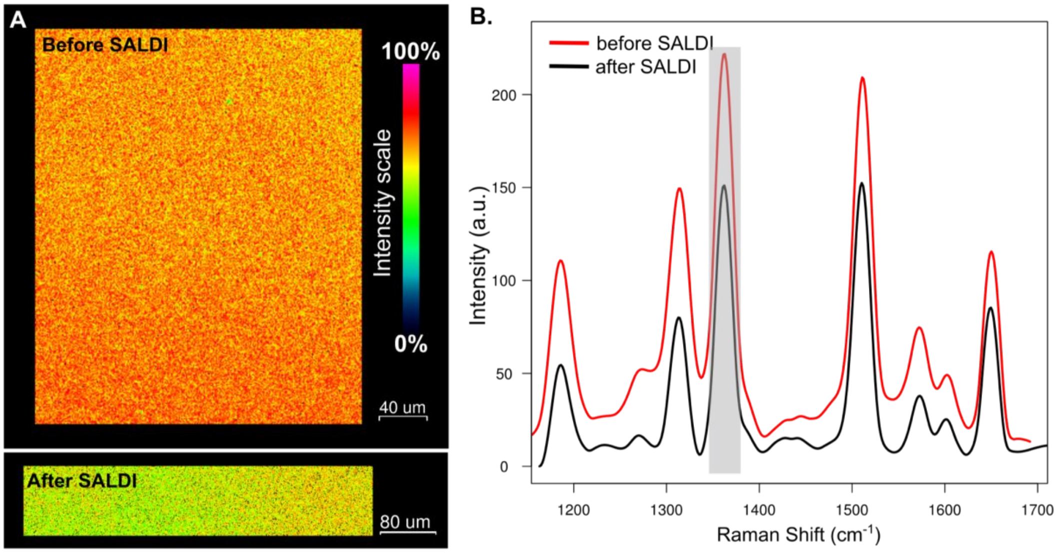

Figure S8. Imaging acquisition order assessment for high resolution (2 µm) SERS maps. A: SERS intensity maps of the 1362 cm^{-1} band collected before and after SALDI measurement. **B:** average spectrum of the SERS maps from (A). Grey rectangle highlights the 1362 cm^{-1} band.

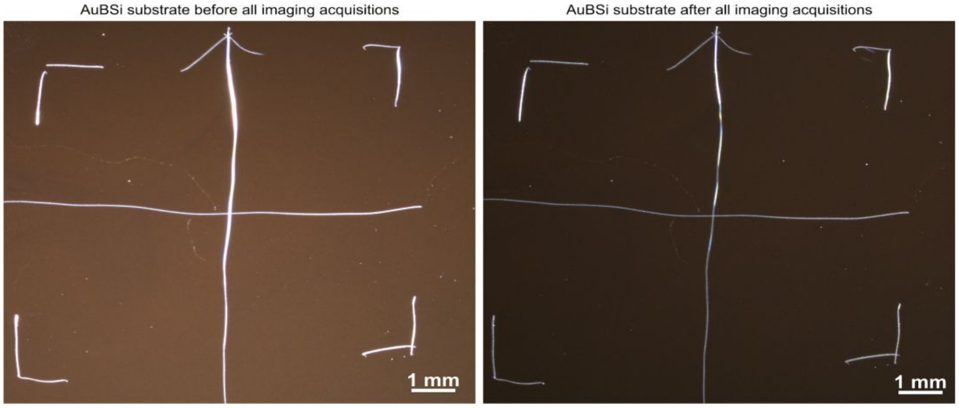

Figure S9. Optical images of the AuBSi substrate before and after imaging acquisitions. The substrate was removed from the Rhodamine 6G solution, dipped 3 times in milli-Q water and then dried at room temperature for 30 minutes.

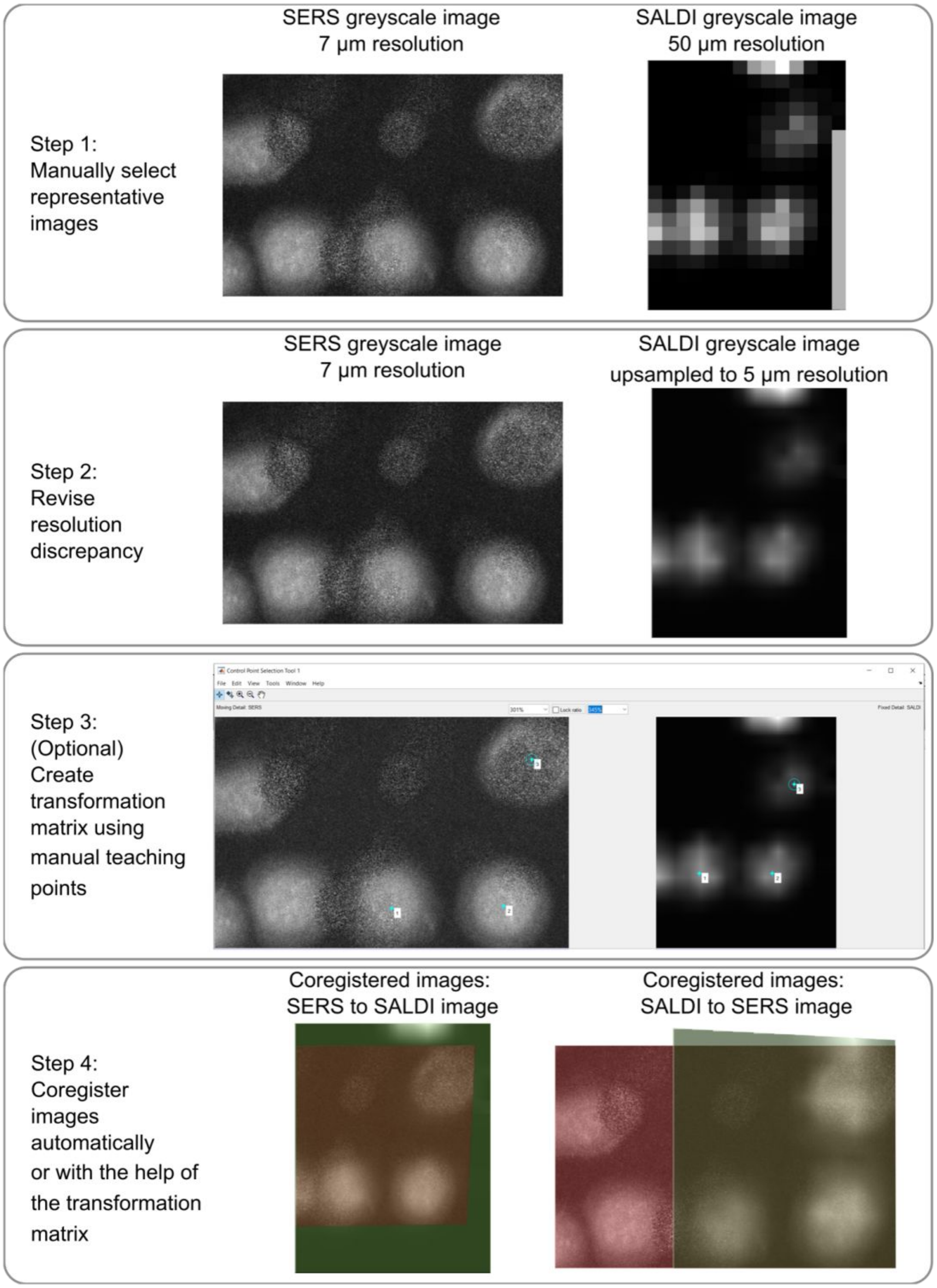

Figure S10. Coregistration algorithm steps. SERS and SALDI representative images collected from the inkjet printed Raman reporters; measurement area marked with a blue rectangle in Figure S2. The representative image for SALDI is the *m/z* 443.29 ion intensity distribution (green hue) and for SERS is the 1513 cm^{-1} band intensity distribution (red hue).

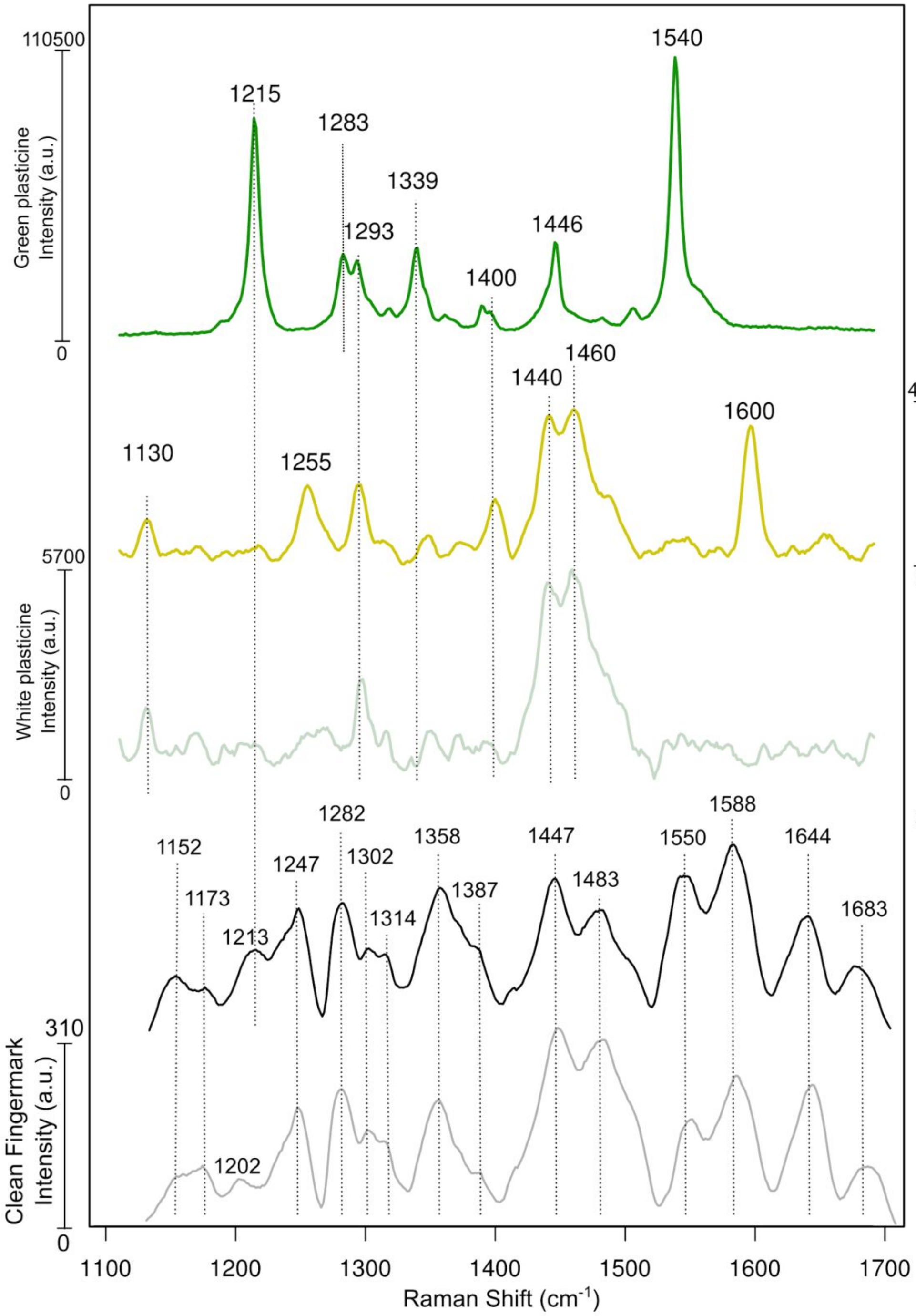

Figure S11. Raman reference spectra collected from white, yellow and green plasticine and average spectra for clean and stained fingermark.

Table S2. **Raman band tentative assignments.** In green we highlighted the bands from the green pigment of the plasticine.

Band (cm^{-1})	Vibration	Assignment	Predominant in	Reference
1152	C-C stretching	carotenoids	both	[2]
1173	C-C stretching	aliphatic carbon chains	both	[3]
1202	ring breathing	tyrosine	clean	[2]
1213	C-C_6H_5 stretching; Stretching of C-N	tyrosine and phenylalanine; no assignment	stained	[4]
1215	ring C-C stretching	Pigment Green 7 (PG7), Cu-Phthalocyanine	green plasticine	[5,6]
1247	β-sheet	Amide III	both	[7]
1282	amide III, CH_2 wagging	glycine, proline	both	[8]
1302	twisting CH_2, dCH_3, dCH_2	lipids, aliphatic carbon chains	both	[23]
1314	-	collagen, lipids or guanine	both	[4]
1358	C-H deformation; CH_2 deformation	tryptophan, guanine; aliphatic carbon chains	both	[3, 7, 8]
1387	CH_3 band	squalene	stained	[2, 4]
1447	$\delta(CH_2/CH_3)$	lipids	both	[2]
1483	ring breathing	guanine, adenosine (ring breathing modes in the DNA bases)	both	[4]
1540	C-N_m-C stretching vibration	Pigment Green 7 (PG7), Cu-phthalocyanine	green plasticine	[5,6]
1551	indole ring vibration	tryptophan	both	[2]
1587	aromatic ring vibrations, C=C	phenylalanine, tyrosine, tryptophan	both	[9]
1646	amide I	alpha-helix proteins	both	[3, 7]
1683	amide I	disordered structure; non hydrogen bonded	both	[4]

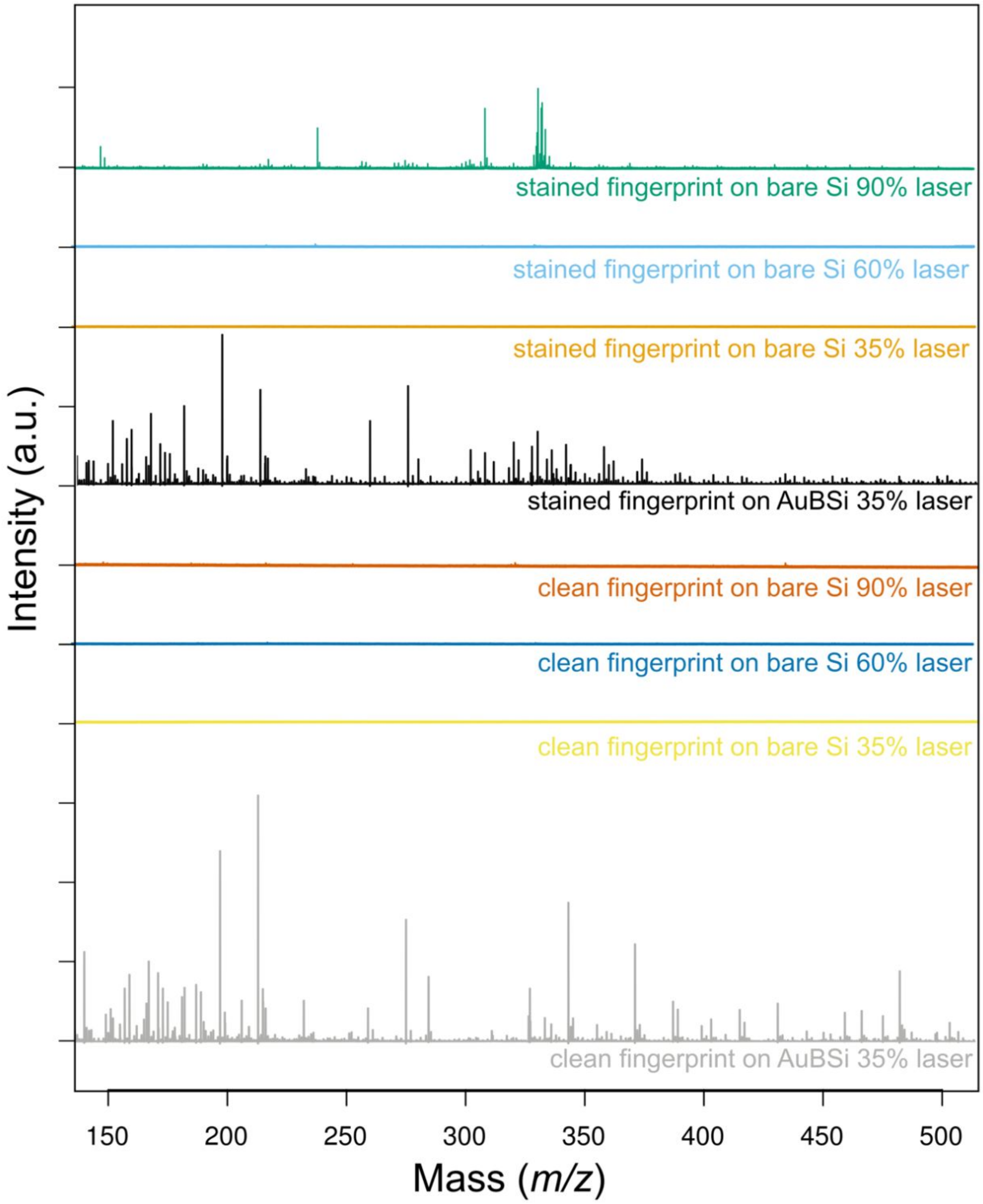

Figure S12. Comparison of clean and stained fingermark spectra collected from a bare silicon wafer and our AuBSi at three different laser power settings (35%, 60%, 90%).

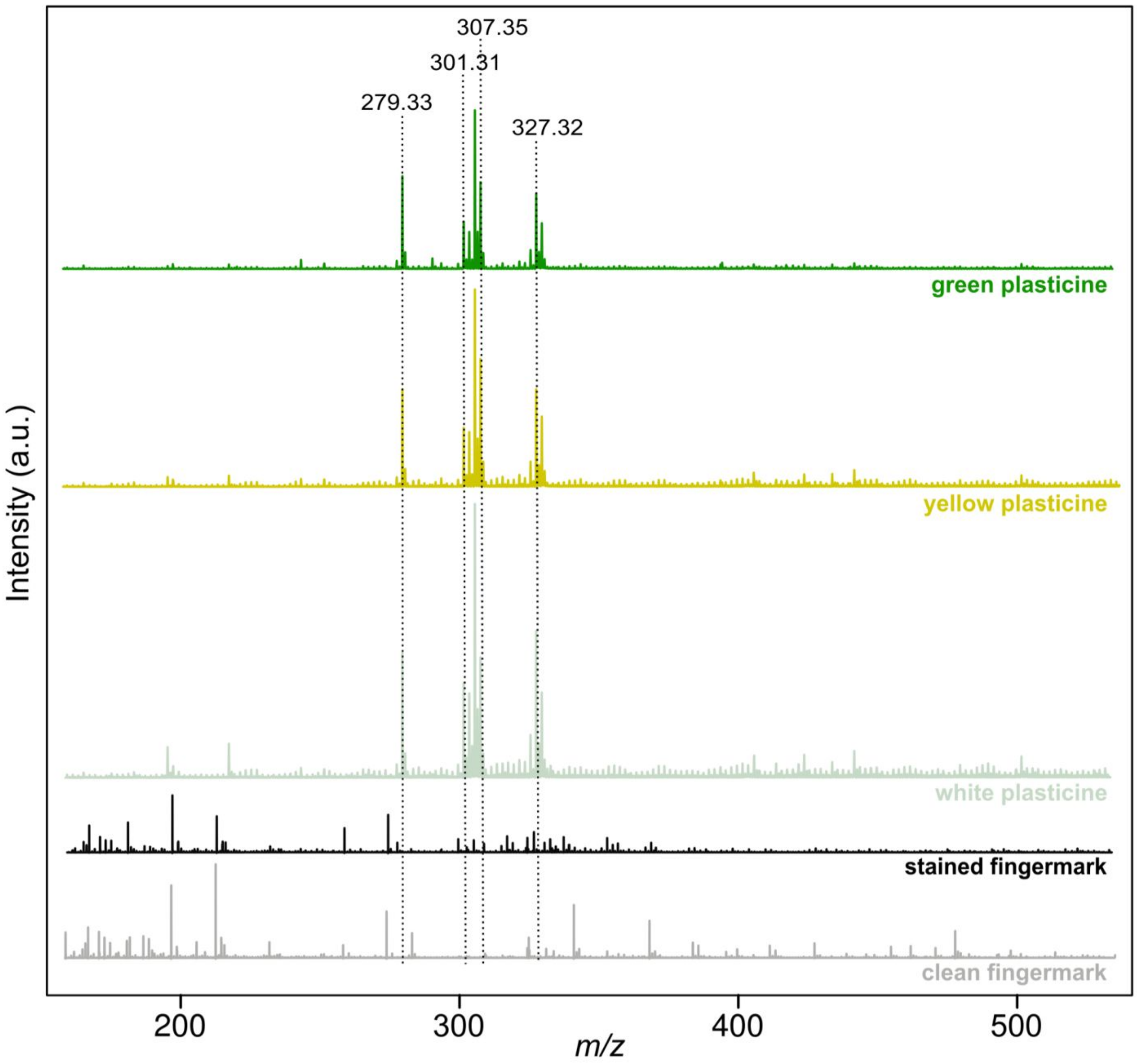

Figure S13. Average spectra of clean (in grey) and stained (in black) fingermarks and reference spectra of white, yellow, and green plasticine (in light grey, yellow and green). The *m/z* features specific to plasticine are marked with a dashed line.

Table S3. Putative identification of ions detected from the clean and stained fingermarks

m/z experimental	*m/z* theoretical	Error (Δ ppm)	Formula	Adduct	Predominant in	Compound name
174.9692	175.0149	261	$C_{13}H_{19}$	$[M+H-H_2O]^+$	Both	Cholesterol fragment
216.1206	216.1025	83	-	$[M+H]^+$	Both	Melatonin fragment[10] Or carnitines (*e.g.* Propenoylcarnitine, Hydroxypropionylcarnitine)
279.3366	278.2974	114	$C_{20}H_{38}$	$[M+H]^+$	Stained	Plasticine - hydrocarbon
301.3065	301.3065	229	$C_{17}H_{33}O_4$	$[M+H]^+$	Stained	Plasticine - monoacylglycerols-MG(14:1)
305.3402	305.2456	309	$C_{18}H_{34}O_2$	$[M+Na]^+$	Stained	Oleic acid from white oil in plasticine
307.3545	307.3545	178	$C_{21}H_{39}O$	[M+H-H2O]$^+$	Stained	Plasticine - wax ester WE(21:1)
327.3051	327.3258	63	$C_{21}H_{43}O_2$	$[M+H]^+$	Stained	Plasticine - wax ester WE(21:0)
329.3175	329.2686	148	$C_{19}H_{37}O_4$	$[M+H]^+$	Stained	Plasticine - monoacylglycerols-MG(17:0)
431.1189	431.0982	48	$C_{16}H_{24}O_{10}S$	$[M+Na]^+$	Both	4-Methoxybenzenepropanol 1-(2-9sulfoglucoside)[10] or probably exogenous, as flavonoids (*e.g.* Auresin) or pyranoxanthones (*e.g.* Silyhermin)
589.4689	589.4802	19	$C_{35}H_{66}O_5$	$[M+Na]^+$	Both	DAG(32:1)
843.8181	843.7776	48	$C_{53}H_{104}O_5$	$[M+Na]^+$	Both	TAG(50:0)
851.7725	851.7463	31	$C_{54}H_{100}O_5$	$[M+Na]^+$	Both	TAG(51:3)
867.7721	867.7412	36	$C_{54}H_{100}O_6$	$[M+Na]^+$	Both	TAG(51:2)
911.8608	911.8402	23	$C_{58}H_{112}O_5$	$[M+Na]^+$	Both	TAG(55:1)

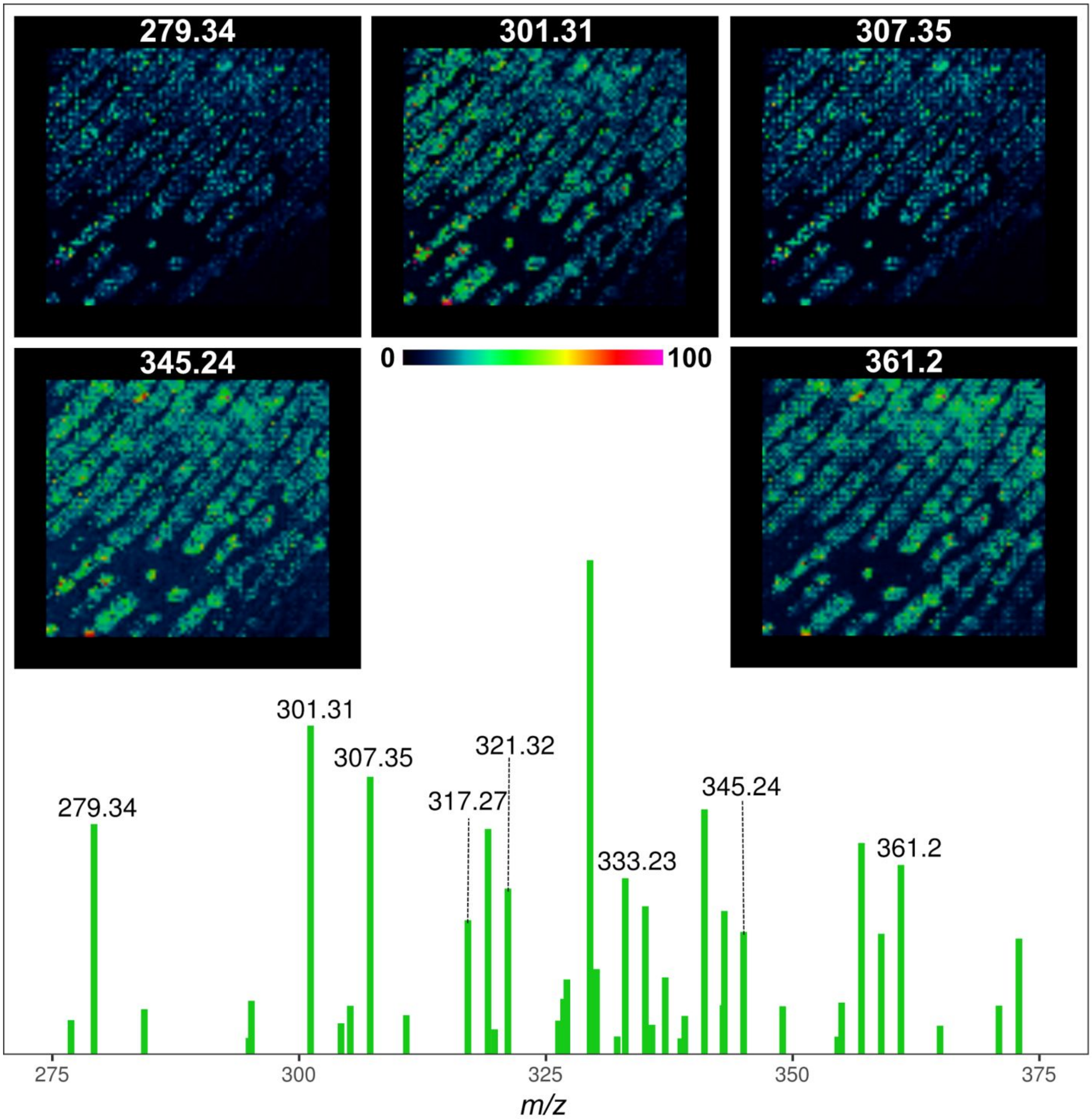

Figure S14. Average spectra of SERS-to-SALDI-MS cluster 1 in the range *m/z* 275-375; Labelled ions appear only in this cluster, which was associated with signal from the stain. The *m/z* images illustrate the distribution of the most intense stain-associated ions. Measurement area 4.4 x 4 mm^2 at 50 µm lateral resolution.

REFERENCES

(1) Lafuente, M.; Pellejero, I.; Clemente, A.; Urbiztondo, M. A.; Mallada, R.; Reinoso, S.; Pina, M. P.; Gandía, L. M. In Situ Synthesis of SERS-Active Au@POM Nanostructures in a Microfluidic Device for Real-Time Detection of Water Pollutants. *ACS Applied Materials and Interfaces* **2020**, *12* (32), 36458–36467. https://doi.org/10.1021/acsami.0c06725.

(2) Andersson, P. O.; Lejon, C.; Mikaelsson, T.; Landström, L. Towards Fingermark Dating: A Raman Spectroscopy Proof-of-Concept Study. *ChemistryOpen* **2017**, *6* (6), 706–709. https://doi.org/10.1002/open.201700129.

(3) Dorakumbura, B. N.; Boseley, R. E.; Becker, T.; Martin, D. E.; Richter, A.; Tobin, M. J.; van Bronswjik, W.; Vongsvivut, J.; Hackett, M. J.; Lewis, S. W. Revealing the Spatial Distribution of Chemical Species within Latent Fingermarks Using Vibrational Spectroscopy. *Analyst* **2018**, *143* (17), 4027–4039. https://doi.org/10.1039/c7an01615h.

(4) Movasaghi, Z.; Rehman, S.; Rehman, I. U. Raman Spectroscopy of Biological Tissues. *Applied Spectroscopy Reviews* **2007**, *42* (5), 493–541. https://doi.org/10.1080/05704920701551530.

(5) Scherrer, N. C.; Stefan, Z.; Francoise, D.; Annette, F.; Renate, K. Synthetic Organic Pigments of the 20th and 21st Century Relevant to Artist's Paints: Raman Spectra Reference Collection. *Spectrochimica Acta - Part A: Molecular and Biomolecular Spectroscopy* **2009**, *73* (3), 505–524. https://doi.org/10.1016/j.saa.2008.11.029.

(6) el Mendili, Y.; Vaitkus, A.; Merkys, A.; Gražulis, S.; Chateigner, D.; Mathevet, F.; Gascoin, S.; Petit, S.; Bardeau, J. F.; Zanatta, M.; Secchi, M.; Mariotto, G.; Kumar, A.; Cassetta, M.; Lutterotti, L.; Borovin, E.; Orberger, B.; Simon, P.; Hehlen, B.; le Guen, M. Raman Open Database: First Interconnected Raman–X-Ray Diffraction Open-Access Resource for Material Identification. *Journal of Applied Crystallography* **2019**, *52* (3), 618–625. https://doi.org/10.1107/S1600576719004229.

(7) Cennamo, G.; Montorio, D.; Morra, V. B.; Criscuolo, C.; Lanzillo, R.; Salvatore, E.; Camerlingo, C.; Lisitskiy, M.; Delfino, I.; Portaccio, M.; Lepore, M. Surface-Enhanced Raman Spectroscopy of Tears: Toward a Diagnostic Tool for Neurodegenerative Disease Identification. *Journal of Biomedical Optics* **2020**, *25* (08), 1. https://doi.org/10.1117/1.jbo.25.8.087002.

(8) Kolhatkar, G.; Parisien, C.; Ruediger, A.; Muehlethaler, C. Latent Fingermark Imaging by Single-Metal Deposition of Gold Nanoparticles and Surface Enhanced Raman Spectroscopy. *Frontiers in Chemistry* **2019**, *7* (JUN), 1–8. https://doi.org/10.3389/fchem.2019.00440.

(9) Liu, X.; Lu, L.; Li, Z.; Song, W.; Lu, Y.; Mao, Z.; Zhao, B. Detection of Protein Deposition within Latent Fingerprints by Surface-Enhanced Raman Spectroscopy Imaging. *Nanoscale* **2012**, *4* (7), 2333. https://doi.org/10.1039/c2nr12030e.

(10) Brunmair, J.; Niederstaetter, L.; Neuditschko, B.; Bileck, A.; Slany, A.; Janker, L.; Feuerstein, M. L.; Langbauer, C.; Gotsmy, M.; Zanghellini, J.; Meier-Menches, S. M.; Gerner, C. Finger Sweat Analysis Enables Short Interval Metabolic Biomonitoring in Humans. *bioRxiv Biochemistry* **2020**, 2020.11.06.369355.
